# Assessing proliferation, cell-cycle arrest and apoptotic end points in human buccal punch biopsies for use as pharmacodynamic biomarkers in drug development

**DOI:** 10.1038/sj.bjc.6602686

**Published:** 2005-07-05

**Authors:** D R Camidge, M N Pemberton, J W Growcott, D Johnstone, P J Laud, J R Foster, K J Randall, A M Hughes

**Affiliations:** 1 Edinburgh Cancer Centre, Western General Hospital, Edinburgh EH4 2XU, UK; 2 University Dental Hospital of Manchester, Manchester M15 6FH, UK; 3 AstraZeneca, Alderley Park, Macclesfield, Cheshire SK10 4TG, UK

**Keywords:** buccal biopsy, proliferation, apoptosis, tolerability, feasibility, variability

## Abstract

Easily accessible normal tissues expressing the same molecular site(s) of drug action as malignant tissue offer an enhanced potential for early proof of anticancer drug mechanism and estimation of the biologically effective dose. Studies were undertaken in healthy male volunteers to assess the tolerability of single and multiple (four in 24 h) 3 mm punch biopsies of the buccal mucosa, and to determine the feasibility of detecting and quantifying a range of proliferation, cell-cycle arrest and apoptosis markers by immunohistochemistry (IHC) for use as potential pharmacodynamic (PD) end points. The biopsy procedure was well tolerated with 100% of volunteers stating that they would undergo single (*n*=10) and multiple (*n*=12) biopsies again. Total retinoblastoma protein (pRb), phosphorylated pRb (phospho-pRb), total p27, phosphorylated p27 (phospho-p27), phosphorylated-histone H3 (phospho-HH3), p21, p53, Cyclin A, Cyclin E, Ki67 all produced good signal detection, but M30, cleaved caspase 3 and terminal deoxynucleotidyl transferase-mediated dUTP-biotin nick end labelling did not. Total pRb, phospho-pRb, total p27 and phospho-p27 were quantified further in a multiple biopsy study to allow components of variability to be addressed to inform future sizing decisions on intervention studies. Neither site of biopsy within the oral cavity, nor the nominal time of biopsy had any significant impact on any of the four markers expression levels. Inter- and intrasubject coefficients of variation (CVs) that could be used to size future intervention studies for pRb, phospho-pRb, total p27 and phospho-p27 were 14, 19, 18 and 16%; and 18, 29, 25 and 19%, respectively. In conclusion, quantitation of such markers in 3 mm buccal punch biopsies would be suitable to explore as PD end points within intervention studies of drugs acting on these pathways.

Linking the molecular effects of drug exposure to the mechanisms of either efficacy or toxicity of the drug would improve early oncology drug development. While pharmacodynamic (PD) effects derived directly from diseased/dysfuntional tissue are likely to relate most closely to clinically relevant outcomes (Baselga, 2003), tissue heterogeneity and tissue accessibility may restrict this approach. PD biomarkers in relatively homogenous, more accessible normal tissues may offer an easier approach to establishing proof of drug mechanism and to make an early assessment of exposure–response relationships.

Normal tissue-based PD approaches have already been used with success during the clinical development of anticancer drugs acting on biomarkers present within peripheral blood mononuclear cells (Cohen *et al*, 2003; Peralba *et al*, 2003), exfoliated buccal squames (van de Vaart *et al*, 2000; Adjei *et al*, 2003) and punch biopsies of the skin (Albanell *et al*, 2002; Malik *et al*, 2003; Vanhoefer *et al*, 2004). The choice of normal tissue for a given drug study is likely to depend on a number of different factors, including the level and variability of expression of the biomarker of interest. Literature reports suggest that the buccal mucosa has a high baseline proliferation index (Jordan *et al*, 1998; Hirota *et al*, 2002; Kurokawa *et al*, 2003), making it a potentially attractive tissue for assessing antiproliferative end points. Unfortunately, these indices are often obtained from individuals undergoing follow up for various oral pathologies, therefore field effects skewing these ‘normal’ values cannot be ruled out. In addition, there are few published details on the tolerability of repeated buccal biopsies as would be required during time-course PD assessments, or of the baseline variability components of key biomarkers to inform sizing decisions for drug intervention studies designed to impact these markers.

## MATERIALS AND METHODS

### Study design and healthy volunteer recruitment

Two separate open nonrandomised buccal biopsy studies (Study A and Study B) involving 10 and 12 healthy male volunteers, respectively, were approved by an independent ethics committee and conducted in full accordance with the Declaration of Helsinki and the International Committee on Harmonisation's guidelines on Good Clinical Practice. Study-specific exclusion criteria included the use of tobacco-based products within 1 year of the start of the study.

Study A was designed to address the tolerability, in practice, of performing single buccal biopsies; the tolerability, in theory, of performing multiple buccal biopsies; and the feasibility of measuring a range of different proliferation, cell-cycle arrest and apoptotic biomarkers in buccal biopsy specimens by immunohistochemistry (IHC). Study B was designed to address the tolerability of multiple buccal biopsies in practice and the variability components of four key biomarkers. The volunteers underwent a single buccal biopsy in Study A and four separate buccal biopsy procedures over a 24-h period in Study B. The timings of biopsies for each subject were expressed as both actual timings according to the 24-h clock and as nominal timings (4, 8 and 24 h) relative to the timing of the first biopsy (0 h).

The volunteers returned 24 h (±30 min) post-completion of all biopsy procedures (Study A), and again at 14 days (±2 days) (Studies A and B) for tolerability assessments and inspection of the biopsy site(s). Any adverse events reported by the subjects were graded according to the National Cancer Institute Common Terminology Criteria (Version 3, March 2003).

### Biopsy procedure and tissue handling

All buccal biopsies were performed under aseptic conditions by, or under the direct supervision of, a consultant on the UK specialist list for Oral Medicine. After local anaesthesia was achieved with approximately 1 ml of 2% lidocaine with 1 : 80 000 epinephrine (AstraZeneca, Macclesfield, UK), a 3 mm biopsy punch (Stiefel Laboratories Limited, Buckinghamshire, UK) was used to obtain a core from the buccal mucosa of either the left or right cheek near the level of the occlusal line, and then immersed in 10% neutral-buffered formalin. When multiple biopsies were obtained, all four quadrants of the mouth were utilised, with the order of location of the four biopsies in each subject (front/back, left/right) being randomly determined at the commencement of their entry into the study.

### IHC and signal quantification

Following a uniform fixation period of as close to 48 h as possible, the biopsy specimens were embedded in paraffin. Serial sections (4 *μ*m) were cut, and dried on slides at 37°C overnight.

Dewaxed sections were subjected to heat-induced epitope retrieval in a microwave pressure cooker containing 0.01 M citrate buffer, pH 6.0 for 2 min at 120°C.

Immunohistochemistry staining was performed using a Lab Vision Autostainer (Lab Vision [UK] Ltd, Newmarket, UK).

Endogenous hydrogen peroxidase activity was quenched by incubation in 3% aqueous H_2_O_2_ for 10 min, followed by a 20 min incubation in serum-free protein block (X0909, DakoCytomation Ltd., Ely, Cambridgeshire, UK). Primary antibody, diluted in tris-buffered saline with 0.1% Tween 20, was applied for 60 min. Detection was achieved using Dako's Envision+Horse Radish Peroxidase-labelled polymer system (K4007 for mouse primary antibodies, K4011 for rabbit primary antibodies) with 3,3 diaminobenzidine tetrahydrochloride as the chromogen (DakoCytomation Ltd, Ely, Cambridgeshire, UK). All incubations were at room temperature, and sections were washed thoroughly in tris-buffered saline between each step. Sections were counterstained with Carazzi's haematoxylin before being dehydrated, cleared and mounted in Histomount® (Raymond A Lamb Ltd, Eastbourne, UK). Terminal deoxynucleotidyl transferase-mediated dUTP nick end labeling (TUNEL) for the detection of apoptotic cells was performed according to the kit (Roche Diagnostics GmbH, Penzberg, Germany) using the Ventana Discovery platform (Ventana Medical Systems S.A., Illkirch Cedex, France). Details of the antibodies and techniques used for each marker are shown in Table 1.

Sections from previously characterised xenografts were used as positive controls. Suitable isotype/Ig fractions were used to stain negative control buccal biopsy sections. Antibodies were validated by preadsorption of the antiserum with the peptide antigen used to generate the antibody, where available, looking for complete ablation of any antibody-binding signal. Phospho-specific antibodies were further appraised by looking for complete signal abolition following pre-incubation of sections with alkaline phosphatase (713 023 Roche Diagnostics GmbH, Penzberg, Germany).

Image analysis and quantification was performed with the Zeiss KS400 system (Imaging Associates, Bicester, Oxfordshire, UK) using a bespoke macro programme linked to a Leica DMRB microscope. Care was taken not to overlap fields of view to ensure that cells were not counted twice. The number of positive nuclei was expressed per mm of basement membrane (BM) as a unit length labelling index (ULLI) (Monticello *et al*, 1990).

### Statistical analysis

Biomarker data were log-transformed prior to analysis and compared to untransformed data. The intersubject variability of those IHC markers that were assessed within Study A but not within Study B was calculated. Coefficient of variation (CV) was calculated as square root (e(*s*^2^)−1) × 100%, where *s* was the standard deviation (s.d.) of the log-transformed data.

The primary objective of Study B was to assess the intrasubject variability of total retinoblastoma protein (pRb), phosphorylated pRb (phospho-pRb), total p27 and phosphorylated p27 (phospho-p27). The secondary objectives of Study B were to assess the intersubject variability, the evidence for any effect of biopsy site or of nominal or actual biopsy time on these marker levels; and to assess the relationship between total and phospho-pRb, and between total and phospho-p27, as ratios. Components of variation were estimated from an analysis of variance (ANOVA) model, using an *F*-test calculated from proc mixed within SAS. The models constructed allowed for the effects of nominal time, actual time and subject. Times were fitted as fixed effects, with subjects fitted as random effects. Statistical significance was assumed to be when *P*<0.05.

No inferential statistical analyses were undertaken on data from the tolerability assessments in Studies A or B.

## RESULTS

**Figure 1:**
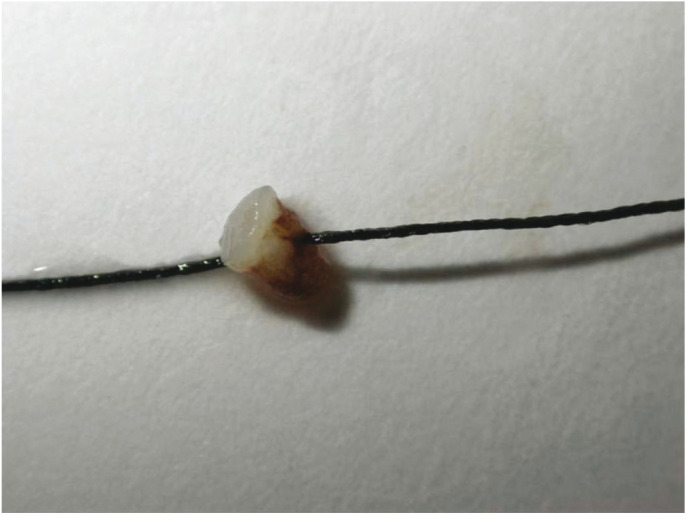
Human buccal biopsy (3 mm), postfixation, with suture *in situ* for ease of handling.

The mean ages of the volunteers in Studies A and B were 45.3 and 42.6 years, respectively. Each biopsy procedure took between 10 and 20 min. Actual biopsy times varied by as much as 90 min for the same nominal time point between individuals. A suture passed through the base of the biopsy pedicle prior to devascularisation (Figure 1) was found to facilitate handling of the specimen and minimise crush artefact. In addition, the hole left by the suture assisted in the orientation of the specimen during subsequent analyses.

### Tolerability and adverse events

The specific questions asked, and the answers that the subjects gave, relating to overall tolerability in terms of their willingness to undergo repeat biopsies, based on their actual and hypothetical experiences in Study A, are presented in Table 2.

In Study B, based on their actual experiences of four biopsies in 24 h, all 12 subjects answered ‘YES’ to the overall tolerability question: ‘Would you undergo the same procedure again?’

The adverse events recorded during Studies A and B, considered causally related to the study procedures, are presented in Table 3.

### Feasibility of detecting proliferation and apoptosis markers in buccal mucosa

**Figure 2:**
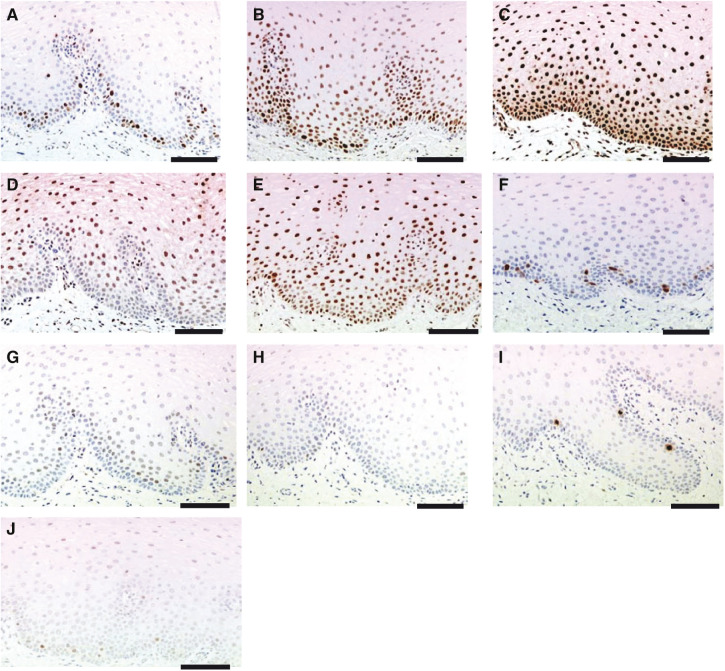
Representative sections of buccal biopsy tissues stained for (**A**) Ki67, (**B**) total pRb, (**C**) phospho-pRb, (**D**) total p27, (**E**) phospho-p27, (**F**) cyclin A, (**G**) p21, (**H**) p53, (**I**) phospho-HH3 and (**J**) cyclin E. The solid horizontal line on each picture represents a scale of 100 *μ*m.

Signals detectable on IHC in buccal biopsies compatible with the expected patterns of cellular staining were obtained for Ki67, total pRb, phospho-pRb, total p27, phospho-p27, Cyclin E, p21, p53, phosphorylated-histone H3 (phospho-HH3) and Cyclin A (Figure 2A–J). The apoptotic markers M30 and cleaved caspase 3 worked well in control xenograft tissue, but produced no detectable signals in the buccal mucosa. Signals were obtained in buccal mucosa with TUNEL, but this technique appeared relatively insensitive to apoptosis in our hands, failing to detect some overtly apoptosing cells (data not shown).

### Components of variability

**Figure 3:**
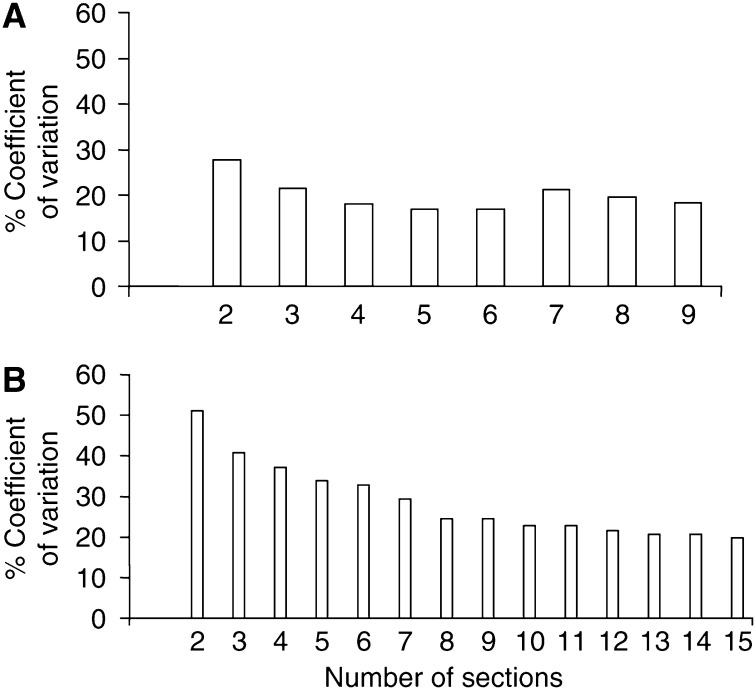
Effect of number of stepped sections counted per block on the variability of marker expression. Each histogram represents the CV derived from the expression levels of (**A**) phospho-pRb and (**B**) phospho-HH3 based on sampling from 2 to 9 and 2 to 15 serial sections, respectively, of buccal biopsy tissue.

Multiple, stepped sections (⩾ every fifth section) from each biopsy in Study A were stained and analysed to determine the optimum number of sections required for each marker. Since stepped sections were employed, with an interval of at least 16 *μ*m, a single cell was not counted more than once, in effect increasing the length of buccal mucosa measured for each marker. The optimum number of sections was assumed to be where the graph of CV plotted against the number of sections leveled out, that is, the point beyond which increasing the number of sections did not reduce the variability further. For markers exhibiting relatively high levels of expression, such as phospho-pRb, only five sections needed to be measured before the variability plateaued, but for a marker exhibiting a lower relative frequency of expression, such as phospho-histone H3, the number of sections required increased to 11 (Figure 3). For reasons of practicality, five stepped sections were quantified for all markers within Studies A and B.

Exploration of the data for all transformed markers revealed them to be log-normally distributed. Quantitative scores were generated from the biopsies within Study A for the following markers: total pRb, phospho-pRb, total p27, phospho-p27, phospho-HH3, p21, p53, Cyclin A, Cyclin E, Ki67 and TUNEL. The summary statistics for the markers that were not assessed further in Study B are presented in Table 4. For those markers with low signal levels, the estimates of variability could well be improved upon if more sections per block had been counted.

In Study B, four markers (total pRb, phospho-pRb, total p27 and phospho-p27) were quantified to allow components of variability to be addressed in detail. With the exception of total pRb, sections were quantified from each of the 12 subjects in Study B. In three subjects, it was found that the staining for total pRb was punctate, intense and extranuclear. The reason for this deviation from the normally accepted nuclear pattern of staining was unknown, but the data on total pRb from these three subjects in Study B were not included in the subsequent analysis.

**Figure 4:**
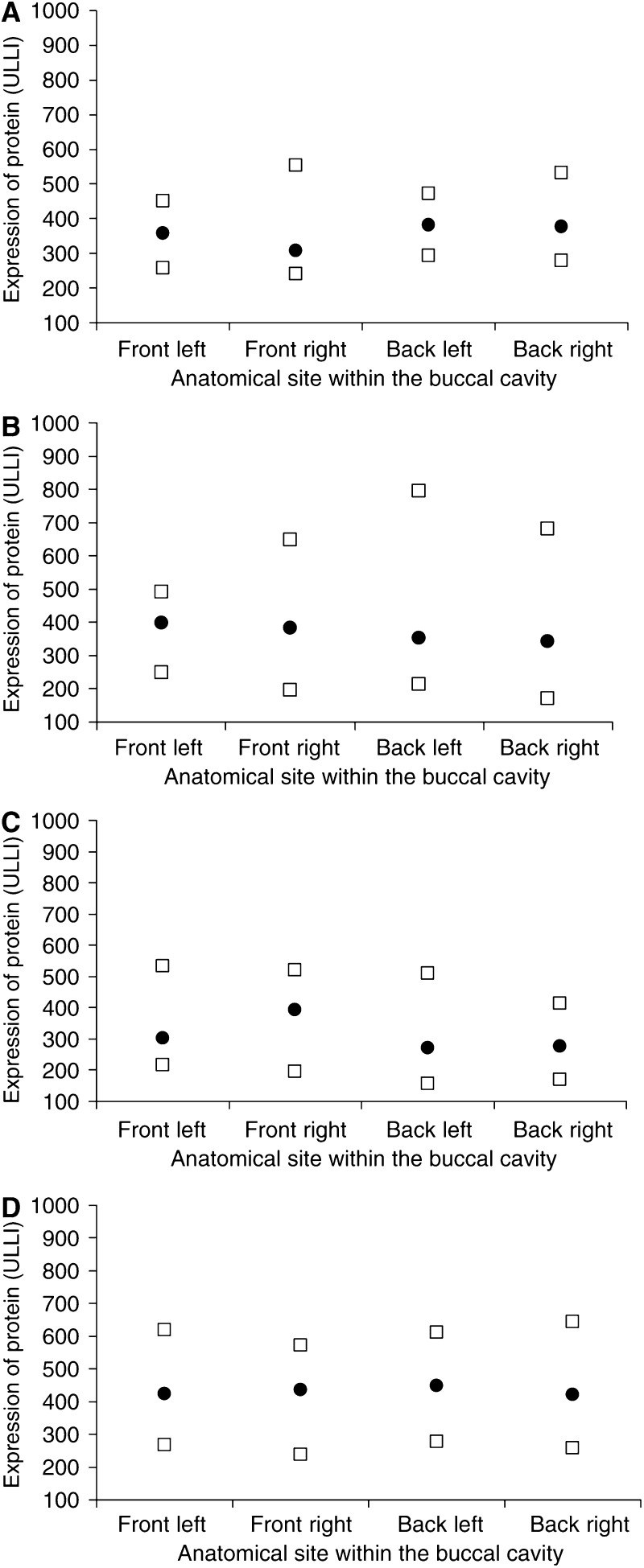
Lack of effect of anatomical site of buccal biopsy on expression levels of markers (**A**) total pRb (*n*=9), (**B**) phospho-pRb (*n*=12), (**C**) total p27 (*n*=12), (**D**) phospho-p27 (*n*=12). Solid symbols (▪) represent the geometric means in buccal biopsies taken from either the front-left, front-right, back-left or back-right regions of the oral cavity. Open symbols (□) represent the maxima and minima associated with the same markers at each anatomical site.

**Figure 5:**
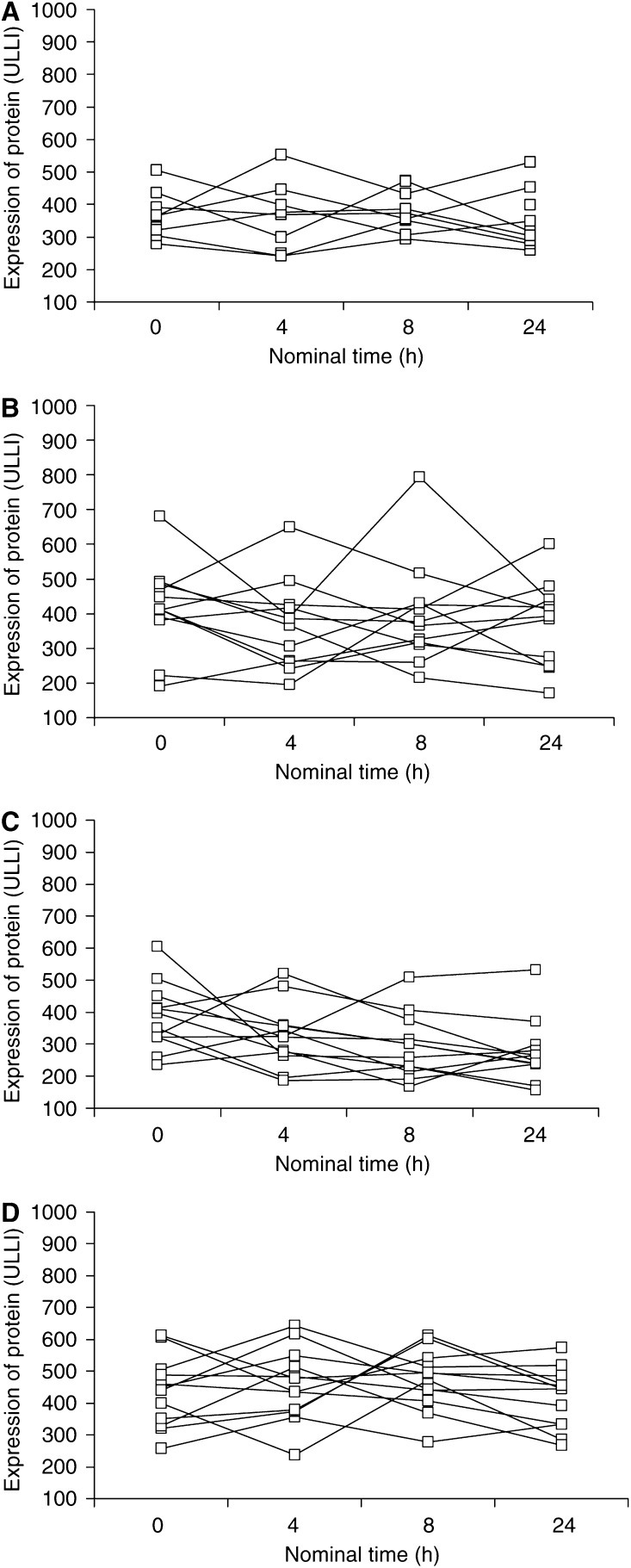
Lack of effect of nominal time of buccal biopsy on expression levels of markers (**A**) total pRb (*n*=9), (**B**) phospho-pRb (*n*=12), (**C**) total p27 (*n*=12), (**D**) phospho-p27 (*n*=12), taken from each subject at nominal times of 0, 4, 8 and 12 h.

There was no discernible pattern of biopsy site within the oral cavity (front/back, left/right) on visual inspection of the group geometric mean expression levels of these markers (Figure 4A–D), or within individuals (data not shown). There was also no statistically significant effect (*P*>0.05) on either the group geometric mean expression levels of nominal biopsy time (Table 5) or of actual time (data not shown), or of a consistently discernible pattern within individuals indicating an effect of nominal biopsy time (Figure 5A–D) or of actual time (data not shown). The ANOVA model selected allowed for the effects of nominal time and subject. The effect of actual time was also considered initially, but was not included in the final ANOVA model as there was no evidence of such an effect.

## DISCUSSION

Pharmacodynamic markers of drug action derived from easily accessible normal human tissue could assist clinical drug development. The buccal mucosa is one such easily accessible normal tissue. Buccal mucosa-derived cells may be procured through exfoliation by scraping or brushing, or through removal of solid tissue specimens by incisional or punch biopsies (Epstein *et al*, 2002; Oliver *et al*, 2004). Exfoliated buccal squames are easier to acquire but are predominantly derived from the superficial cell layers. As the buccal mucosa is a stratified epithelium with the majority of proliferating cells being present in the layers at, or near to, the BM (cf Figure 2), standard exfoliative techniques, which do not sample from these deeper layers, may limit the usefulness of this approach for assessing PD end points based on inhibition of proliferation. Sampling the full thickness of the mucosa, punch biopsies, offer advantages over incisional biopsies in terms of simplicity, speed, cost and safety (Lynch and Morris, 1990). They also offer size uniformity, which may be important in standardising both the speed of tissue penetration by fixative and the chances of observing rare cellular events.

In studies A and B, single and multiple 3 mm punch biopsies of the buccal mucosa were well tolerated with minimal procedure-related adverse events (Tables 2 and 3), implying that multiple PD assessments over hours as well as days within the same individual would be practicable with these techniques.

To generate meaningful quantitative data for PD assessments, we attempted to optimise and standardise our methods for tissue preparation, IHC and analysis. Our optimised protocol was inevitably a compromise between the requirements of each biomarker and, for key biomarkers in intervention studies in the future, it may be necessary to have marker-specific protocols for tissue preparation and analysis, rather than to adopt a ‘one size fits all’ approach.

Our quantitative IHC revealed a wide range of expression levels between different markers in the buccal mucosa (Tables 4 and 5). In general, proliferation markers with a very low level of expression are unlikely to be useful as end points in antiproliferative drug studies as the anticipated effect of these drugs would be to make such low levels even lower. In contrast, markers of cell-cycle arrest, such as p27, may be expected to increase in the presence of these drugs (Malik *et al*, 2003). Apoptosis, which could also conceivably increase with certain interventions, is such a rare event in the normal buccal mucosa that its usefulness as a potential PD marker is uncertain (Hirota *et al*, 2002; Loro *et al*, 2002; Hague *et al*, 2004).

Progression through the cell cycle can be monitored by assessing levels of pRb and p27 in both their un/hypophosphorylated (pRb and p27), and hyperphosphorylated (phospho-pRb and phospho-p27) states (Buchkovich *et al*, 1989; Vlach *et al*, 1997; Howard *et al*, 2000). Antibodies directed against total pRb or total p27 are likely to detect both the hyper- and hypo/nonphosphorylated forms of the protein (Buchkovich *et al*, 1989). Elevated levels of p27 induces G1 arrest (Sherr, 2000). To permit hyperphosphorylation of pRb and cell-cycle progression, the inhibitory influence of p27 is removed through hyperphosphorylation, ubiquitination and destruction within the proteasome (Vlach *et al*, 1997; Israels and Israels, 2000). Owing to the high signal levels of total pRb, phospho-pRb, p27 and phospho-p27 found in the buccal mucosa in Study A, and the fact that the expression and activation of these cell-cycle proteins appeared relatively tightly controlled, with the nuclear signal being largely ‘on or off’ (Figure 2B–E) allowing easier automated counting, we chose to explore these markers in more detail in Study B. In addition, there appeared to be an inverse relationship in the expression/activation levels of these proliferation and cell-cycle arrest markers, particularly between total pRb and total p27 (Figures 2B and D). If such ‘mirror image’ signals changed on intervention in opposite but appropriate directions, this would significantly increase an investigator's confidence in attributing any such changes to antiproliferative mechanisms of action (Daneshmand *et al*, 2003). The basal cell layers in which the highest levels of total pRb are seen are also the most actively proliferating, so the total pRb antibody is likely to be detecting cells that have a preponderance of pRb in the hyperphosphorylated state. This is confirmed by the observation that higher levels of phospho-pRb are apparent within the basal cells, compared to the suprabasal cells (Figure 2C). Total pRb levels have not been reported to change significantly throughout the cell cycle *in vitro* (Buchkovich *et al*, 1989), but the marked differences in total pRb expression levels observed between basal and suprabasal cells suggests either this does not hold true for the human buccal mucosa *in vivo*, or that these are not reflecting cell-cycle differences *per se*, but some effect of cellular differentiation as the cells migrate up through the stratified buccal epithelium.

Evidence of circadian rhythms in the normal buccal mucosa has been reported for several different proliferation markers, based on the degree to which data from six biopsies taken over 24 h could be fitted to cosine curves (Bjarnason *et al*, 1999). We found no evidence of such an effect for the markers analysed with respect to time in Study B (Figure 5; Table 5). Either circadian influences do not extend to the markers we used, or if they do exist, given that they would be contained within our estimates of intrasubject variability, their contribution is sufficiently low as to not be significant.

The high signal and low CV of the four markers analysed in Study B (Table 6) make them attractive end points for use in studies that involve drugs affecting these pathways. The information in Table 6 could be used to adequately size either a parallel arm or crossover study, based on estimates of the expected drug effect and the statistical power required to detect such an effect. Ratios of phospho/total pRb or p27, when compared to either the total- or phospho-marker levels alone, did not appear to consistently improve either the intra- or inter-subject CVs for these markers (Table 6). Similarly, the ratios did not reveal any novel patterns in marker expression when analysed by group or individual for either biopsy site or nominal biopsy time effects, compared to the total- or phospho-marker levels alone (data not shown). This may reflect the fact that the antibodies used to detect the total forms of each protein have different binding affinities and potentially different numbers of epitopes compared to the antibodies directed against the hyperphosphorylated forms. Consequently, the total and phospho-signal levels generated by these antibodies for p27 and pRb may not follow a simple linear relationship.

Having established the feasibility and variability of detecting signals for a range of different IHC markers within the buccal mucosa, the next step must be to assess the effect of an antiproliferative drug, or a drug working directly on the pathways that involve these markers, on their expression levels. While it is impossible to state that marker levels and biopsy tolerability in male volunteers will be directly extrapolatable to female subjects or to patients, we believe these data offer a reasonable base from which to start. Evidence of an effect, particularly if supported by opposing changes in proliferation and cell-cycle arrest markers, would act as initial corroboration of the normal tissue-based biomarker approach to proof of drug mechanism and dose/schedule finding described in the introduction to this paper.

## Figures and Tables

**Table 1:** Details of antibodies/techniques used

**Target**	**Supplier**	**Host/type**	**Catalogue number**	**Dilution**	**Detection System**
Total-pRb	CST	Mouse monoclonal	9309	1 : 100	Mouse Envision+
Phospho(S249/T252)-pRb	Biosource	Rabbit polyclonal	44-584	1 : 1000	Rabbit Envision+
Total-p27	RDI	Rabbit polyclonal	P27CabrX	1 : 100	Rabbit Envision+
Phospho(T187)-p27	Upstate	Rabbit polyclonal	06-996	1 : 100	Rabbit Envision+
p53	NovoCastra	Rabbit polyclonal	NCL-p53-CM1	1 : 2500	Rabbit Envision+
Cyclin A	Neomarkers	Mouse monoclonal	MS-1061S1	1 : 40	Mouse Envision+
Cyclin E	NovoCastra	Mouse monoclonal	NCL-CYCLIN E	1 : 20	Mouse Envision+
Ki67	Dako	Mouse monoclonal	M7240	1 : 25	Mouse Envision+
Phospho(S10)-Histone H3	Upstate	Rabbit polyclonal	06–570	1 : 1000	Rabbit Envision+
p21	BD Pharmingen	Mouse monoclonal	610234	1 : 25	Mouse Envision+
Cleaved-Caspase-3	CST	Rabbit polyclonal	9661	1 : 300	Rabbit Envision+
M30	Roche	Mouse monoclonal	2 140 349	1 : 200	Mouse Envision+
TUNEL (*in situ* cell death kit)	Roche	N/A	1 684 817		

pRb=phosphorylated pRb; phospho-p27=phosphorylated p27; TUNEL=terminal deoxynucleotidyl transferase-mediated dUTP-biotin nick end labelling.

CST–New England Biolabs (UK) Ltd, Hitchin, Hertfordshire, UK; Biosource–B-1400 Nivelles, Belgium; RDI–Concord MA 01742-3049 USA; Upstate–Dundee, UK; Novocastra–Newcastle upon Tyne, UK; Neomarkers–Lab Vision (UK) Ltd, Newmarket, Suffolk, UK; Dako–Ely, Cambridgeshire, UK; BD Pharmingen–Cowley, Oxford, UK.

**Table 2:** Tolerability assessment responses from single buccal biopsy study

**Tolerability question**	**Response (% affirmative)**
Would you have the same procedure done again?	100
Would you have, in theory, six biopsies in a day?	80
Would you have, in theory, three biopsies in a day?	90
Would you have, in theory, six biopsies (consecutive days)	90
Would you have, in theory, six biopsies (consecutive weeks)	100

**Table 3:** Adverse events considered to be related to study procedures, by arbitrary subject number, from the single and multiple buccal biopsy studies

**Subject**	**Onset**	**Adverse event**	**Duration (min)**	**Maximum CTC grade**	**Intervention**
1 (Study A)	Immediately after biopsy procedure	Dizziness	5	1	
1 (Study A)	Immediately after biopsy procedure	Biopsy site discomfort	30	1	Paracetamol (same day only)
2 (Study A)	Immediately after biopsy procedure	Feeling hot/abnormal	2	1	
3 (Study B)	3 h, 26 min after third biopsy procedure	Bleeding biopsy site	20	1	
3 (Study B)	3 h, 26 min after third biopsy procedure	Biopsy site discomfort	45	1	Paracetamol (same day only)

**Table 4:** Immunohistochemistry marker quantification in healthy human buccal mucosa (Study A)

**Marker**	**Geometric mean^a^**	**CV (%)^a^**	**Minimum**	**Maximum**
Phospho-HH3	2.14	76.87	0	7.94
p21	42.84	100.44	9.35	126.47
p53	6.74	167.9	0	32.88
Cyclin A	29.53	43.92	12.58	57.33
Cyclin E	55.18	41.93	28.14	86.88
Ki67	37.23	161.45	2.5	108.21
TUNEL	6.67	126.28	0.89	18.29

a When calculating the geometric means and coefficients of variation (CVs) for phospho-HH3 and for p53, because the minimum values for both were zero, the limit of quantitation of the ULLI assay (1.0) was used as the minimum value.

TUNEL=terminal deoxynucleotidyl transferase-mediated dUTP-biotin nick end labelling.

**Table 5:** IHC marker quantification by nominal time point in healthy human buccal mucosa (Study B)

**Marker**	**Nominal time (h)**	**Geometric mean**	**Minimum**	**Maximum**	**95% CI**
Total pRb	0	360.18	278.88	505.32	308.8–420.1
	4	338.41	240.6	553.2	290.2–394.7
	8	377.02	293.24	472.12	323.2–439.7
	24	343.48	257.6	531.28	294.5–400.6
					
Phospho-pRb	0	396.46	190.92	680.74	324.5–484.4
	4	347.91	195.52	649.32	284.7–425.1
	8	374.65	214.47	793.72	306.6–457.8
	24	355.79	169.50	601.75	291.2–434.7
					
Total p27	0	300.19	205.46	414.06	251.6–358.1
	4	311.88	187.14	52.42	261.4–372.2
	8	278.14	168.4	509.41	233.1–331.8
	24	262.44	157.11	532.21	220.0–313.1
					
Phospho-p27	0	421.26	257.51	612.21	374.3–487.1
	4	444.01	238.09	643.44	384.0–513.4
	8	462.34	277.79	611.24	399.8–534.6
	24	405.41	268.02	572.69	350.6–468.8

Phospho-pRb=phosphorylated pRb; phospho-p27=phosphorylated p27.

**Table 6:** Components of variation in IHC marker quantification in healthy human buccal mucosa (Study B)

**Marker**	**Within subject CV (%)**	**Between subject CV (%)**
Phospho-pRb	29	19
Total pRb	18	14
Phospho-pRb /total pRb ratio	30	11
		
Phospho-p27	19	16
Total p27	25	18
Phospho-p27/total p27 ratio	22	24

Phospho-pRb=phosphorylated pRb; phospho-p27=phosphorylated p27.
